# 

**Published:** 1879-10

**Authors:** 


### CONTENTS.

**CONTRIBUTIONS.** PAGE.

Ultraism in Music, BEN. H. PRATT.......171

I Don't Know. THOS K. BEECHER.......174

New York Journalism. AUSBURN TOWNER.......175

My Doctor. J. J. KEYES.......180

**MEDICAL ITEMS AND NEWS.**

Pneumonia—Hemmorrhoids—Phenylated Collodium—Belladonna as an Intestinal Remedy—Goitre—Whooping-cough treated by Tincture Myrrh—Hiccough—Acne—Poultice of Cinchona in Camphar—Nævus—Elastic Crayon of Nitrate of Silver—Hypodermic Injections of Ergotine—Intraliptic Medication—Novel use for Apomorphia—Shock—Epilepsy—Cystitis—Grindelia Robusta in Whooping-cough—Cardiac Dropsy—Retention of Urine—Epilepsy—Hamilton's Tonic—Sweating—Enruesis—Acute Sciatica and Facial Neuralgia—Diarrhœa Mixture—Epithelioma—Koumiss Colds — Anti-Rheumatic Mixture — Diphtheria—Trousseau's Poultice for Rheumatic Arthritis—Cough Mixtures—Chlorate of Potassa Mixture.......183 to 190

**OUR CORRESPONDENCE.**

Dr. Wales on the Thousand Islands—Six Letters with Replies.......191 to 193

**EDITORIAL.**

OVER-EDUCATION.......194
CREMATION.......195

**EDITORIAL CHIT-CHAT.**

Rev. Thos. K. Beecher, D. D.—Removal—Sticking Plasters—Going Abroad—That Book—Mr. Towner and Journalism—That Monument—"Shorty" Hamilton—The Barbed Fence—The Chain Gang—Rev. W. H. H. Murray—Sarah Bernhart—Dick is Satisfied—"Irregular" Consultations.......197 to 198

